# TAT-modified serum albumin nanoparticles for sustained-release of tetramethylpyrazine and improved targeting to spinal cord injury

**DOI:** 10.1186/s12951-020-00766-4

**Published:** 2021-01-21

**Authors:** Yan Lin, Yujie Wan, Xingjie Du, Jian Li, Jun Wei, Ting Li, Chunhong Li, Zhongbing Liu, Meiling Zhou, Zhirong Zhong

**Affiliations:** 1 Department of Pharmaceutical Sciences, School of Pharmacy, Southwest Medical University, Luzhou, 646000 Sichuan China; 2 Department of Pharmacy, The Affiliated Hospital of Southwest Medical University, Luzhou, 646000 Sichuan China

**Keywords:** Human serum albumin, Nanoparticles, Tetramethylpyrazine, Spinal cord injury, Pharmacokinetics

## Abstract

**Background:**

Spinal Cord injury (SCI) is a kind of severe traumatic disease. The inflammatory response is a significant feature after SCI. Tetramethylpyrazine (TMP), a perennial herb of umbelliferae, is an alkaloid extracted from ligustici. TMP can inhibit the production of nitric oxide and reduce the inflammatory response in peripheral tissues. It can be seen that the therapeutic effect of TMP on SCI is worthy of affirmation. TMP has defects such as short half-life and poor water-solubility. In addition, the commonly used dosage forms of TMP include tablets, dropping pills, injections, etc., and its tissue and organ targeting is still a difficult problem to solve. To improve the solubility and targeting of TMP, here, we developed a nanotechnology-based drug delivery system, TMP-loaded nanoparticles modified with HIV trans-activator of transcription (TAT-TMP-NPs).

**Results:**

The nanoparticles prepared in this study has integrated structure. The hemolysis rate of each group is less than 5%, indicating that the target drug delivery system has good safety. The results of in vivo pharmacokinetic studies show that TAT-TMP-NPs improves the bioavailability of TMP. The quantitative results of drug distribution in vivo show that TAT-TMP-NPs is more distributed in spinal cord tissue and had higher tissue targeting ability compared with other treatment groups.

**Conclusions:**

The target drug delivery system can overcome the defect of low solubility of TMP, achieve the targeting ability, and show the further clinical application prospect.
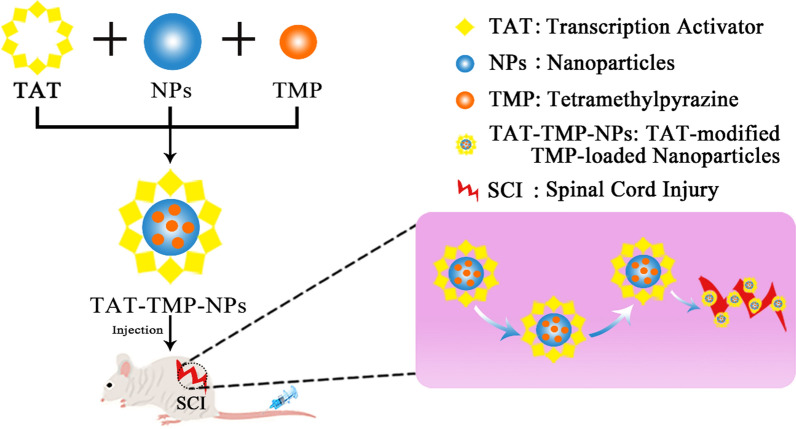

## Introduction

Spinal Cord Injury (SCI) is one of the most common disabled diseases in the world, which is devastating and destructive and it results in incomplete or complete loss of autonomic motor and sensory function [1]. According to the National SCI database, the leading causes of death among patients with SCI since 1973 have been pneumonia and sepsis caused by SCI injuries to respiratory muscles [2, 3]. SCI involves an extremely complex pathological change process, including primary injury and secondary injury. Primary injury is an immediate and irreversible damage; Secondary injury can cause serious and permanent functional deficits [4]. The inflammatory response is an important feature of secondary tissue damage and functional damage [5]. The inflammatory factor diffusion in the early phase after SCI hinders the neurotropic factor infiltration and axonal outgrowth, resulting in paralysis [6]. So, the pathophysiological alterations caused by SCI may last for several years. SCI will not only bring serious physical and psychological damage to patients, but also cause huge economic burden to the whole society. SCI have become a major problem in the medical territory.

Although the development of modern medicine advances the application of trauma surgery and minimally invasive surgery in most clinical departments, both of which may damage tissue, imposing economic and spiritual burdens on the patients [7]. Therefore, we use the traditional Chinese medicine of tetramethylpyrazine (TMP) to treat SCI. The pharmacological effects of TMP mainly include dilating blood vessels, anti-platelet aggregation and improving microcirculation [8]. TMP is also commonly used in clinical ischemic cerebrovascular diseases [9]. Meanwhile, TMP shows protective effects against the spinal cord ischemia-reperfusion injury by reducing apoptosis that resulted from regulating the expression of Bcl-2 and Bax [10]. Moreover, many studies have shown that TMP can down-regulate the nitric oxide production and inhibit inflammation in peripheral tissues, which is the key to promote the functional recovery of acute SCI [11]. Therefore, based on these features of TMP, we speculate that TMP may have beneficial effects on SCI. However, TMP has some disadvantages such as short half-life, poor water-solubility, low bioavailability. To improve the bioavailability of TMP in vivo and enhance the targeting ability to SCI, the key method is to design a new drug delivery system by using modern pharmaceutical preparation technology. It is of great value and significance to SCI patients and society.

In recent years, the application of nanoparticles (NPs) has seen an unprecedented increase in various fields [12]. Nanoparticles can be given by oral administration, skin inhalation, and intravenous injections etc., and finally enter the system circulation [13]. The advantages of nano-based drug delivery systems include the improved therapeutic efficacy and the reduction of side effects [14]. Overall, many of the current categories of available nanomaterials are not acutely toxic but are most likely to have toxic implications following long-term low dose exposures [15]. Therefore, when we prepare nano-preparations, we should give priority to biocompatible materials with low toxicity and easy to be biodegraded such as human serum albumin (HSA).

HSA is a major circulating protein composed of single chain non-glycosylated polypeptides with specific drug binding sites for a variety of endogenous and exogenous compounds [16]. HSA has emerged as a versatile drug delivery platform due to its good biodegradability, non-toxic and non-immunogenic properties [17]. In addition, HSA is mainly phagocytosed by macrophages in the liver, kidney, bone marrow and other tissues after it enters the system circulation, showing good organ-targeting. It was reported that HIV-1 activation of transcription factor (TAT) has trans-membrane transport function and thus TAT could enhance the uptake efficacy of nanoparticles [18]. TAT modified nanoparticles can enter cells through endocytosis or osmosis [19].

Therefore, based on these reports mentioned above, here, we prepared the TMP-loaded nanoparticles modified with TAT (TAT-TMP-NPs) to enhance the bioavailability and targeting ability of TMP to spinal cord injury.

## Results

### Preparation and characterization of HSA nanoparticles

HSA nanoparticles are commonly prepared by the specialized nanotechnological techniques such as desolvation, emulsification, self-assembly, and nanotechnology [20]. In this project, we prepared the HSA nanoparticles by the emulsification-dispersion technique. To assess the effects of different amount of TMP on the particle size, encapsulation efficiency and stability of nanoparticles, we examined the feeding amounts of 0, 10, 20, 30, 40 mg for TMP, and found that the appearance and particle size, encapsulation efficiency of nanoparticles were optimal when the feeding amount of TMP was 30 mg.

**Fig. 1:**
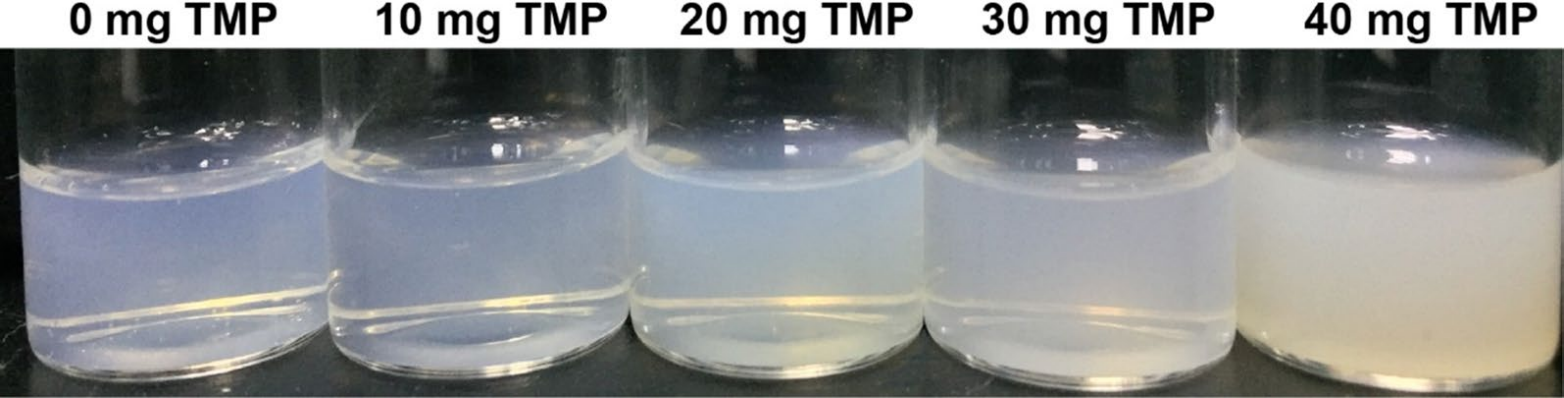
Appearance of TAT-TMP-NPs with different dosages

**Fig. 2:**
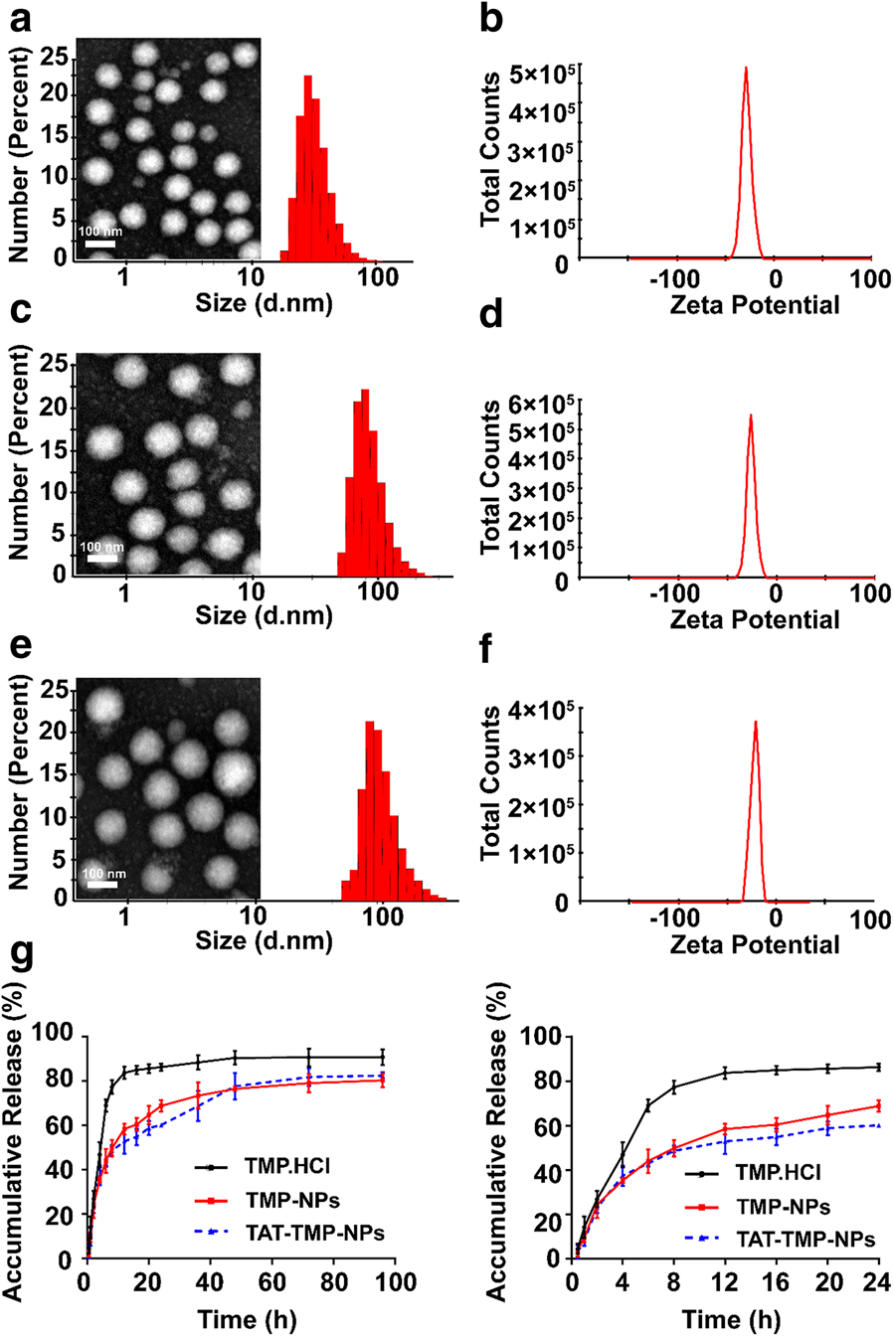
In vitro characterization of Blank-NPs, TMP-NPs and TAT-TMP-NPs. Particle size distribution and TEM images of Blank-NPs (**a**), TMP-NPs (**c**) and TAT--TMP-NPs (**e**). Apparent zeta potential distribution of Blank-NPs (**b**), TMP-NPs (**d**) and TAT-TMP-NPs (**f**) were measured by Malvern Particle Size Analyzer. Results are presented as means ± SD (n = 3). Drug release behaviors of TMP, TMP-NPs and TAT-TMP-NPs were assessed in PBS medium (pH 7.4) at 37 °C and stirred at a speed of 70 rpm for 96 h (**g**). The bar in **a**, **c** and **e** is 100 nm

As shown in Fig. 1; Table 1, the appearance of the prepared HSA nanoparticles was light blue and transparent when the feeding amount of TMP was less than 40 mg. In Fig. 2a, blank-NPs appeared evenly distributed with a particle size of 79.07 ± 0.36 nm (Table 2). The particle sizes of TMP-NPs and TAT-TMP-NPs were much larger (Fig. 2c, e), which increased to 122.57 ± 2.30 nm and 163.93 ± 0.38 nm respectively. Polydispersity index (PDI) of all nanoparticles were smaller than 0.3, indicating the uniformity of nanoparticles.

**Table 1:** Characterization of TAT-TMP-NPs with different dosage

TMP (mg)	Particle size (nm)	Polydispersity index	Zeta potential (mV)	Drug loading (%)	Entrapment efficiency (%)
0	79.07 ± 0.36	0.26 ± 0.01	− 28.50 ± 5.16	–	–
10	96.04 ± 2.77	0.25 ± 0.03	− 22.30 ± 0.56	4.70 ± 0.06	40.50 ± 1.28
20	143.27 ± 0.12	0.25 ± 0.02	− 23.27 ± 0.65	4.90 ± 0.51	62.04 ± 0.08
30	163.93 ± 0.38	0.18 ± 0.01	− 26.10 ± 4.55	8.02 ± 0.12	77.27 ± 1.99
40	258.57 ± 2.60	0.41 ± 0.02	− 20.07 ± 1.01	7.33 ± 1.10	75.70 ± 6.01

**Table 2:** Composition and characterization of nanoparticles

Sample	Particle size (nm)	Polydispersity index	Zeta potential (mV)	Drug loading (%)	Entrapment efficiency (%)
Blank-NPs	79.07 ± 0.36	0.26 ± 0.01	− 28.50 ± 5.16	–	–
TMP-NPs	122.57 ± 2.30	0.13 ± 0.01	− 22.50 ± 4.27	8.36 ± 0.42	75.54 ± 4.02
TAT-TMP-NPs	163.93 ± 0.38	0.18 ± 0.02	− 26.10 ± 4.55	8.02 ± 0.12	77.27 ± 1.99

Moreover, nanoparticles (Fig. 2b, d, f) were negatively charged and the Zeta potential were close to − 30 mV, which indicated that the strong repellent forces among nanoparticles could prevent the aggregation and formed a stable nanoparticles suspension [21]. The transmission electron microscopy also showed that blank-NPs, TMP-NPs, and TAT-TMP-NPs appeared a uniform spherical morphology (Fig. 2a, c, e).

### Drug encapsulation, loading rate and release behavior in vitro

As shown in Table 2, TMP-NPs and TAT-TMP-NPs gave an encapsulation efficiency (EE) of 75.54 ± 4.02% and 77.27 ± 1.99%, and a drug loading (DL) of 8.36 ± 0.42% and 8.02 ± 0.12%, respectively. This result indicated that TMP was effectively loaded into the nanoparticles.

**Fig. 3:**
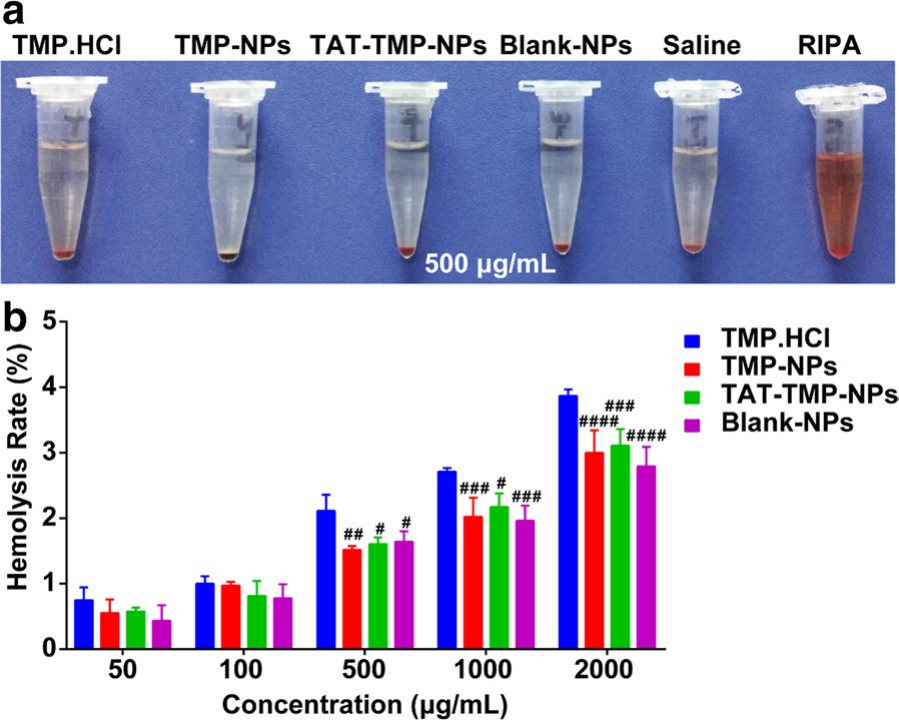
Hemolytic activity of nanoparticles. **a** Red blood cells from healthy SD rats were incubated with 500 µg/mL of concentration of TMP.HCl, TMP-NPs, TAT-TMP-NPs, and Blank-NPs with saline as negative and RIPA as positive controls. **b** Hemolytic activity of different concentrations of various nanoparticle formulations. Values were expressed as mean ± standard deviation (n = 5). ANOVA was used to assess the statistical differences between groups. Symbols represented statistical significance of the labeled groups with group of TMP.HCl (^#^*P* < 0.05, ^##^*P* < 0.01, ^###^*P* < 0.001, ^####^*P* < 0.0001)

The in vitro release behavior of TMP was investigated by a dialysis method, which indicated that both TMP-NPs and TAT-TMP-NPs exhibited a sustained release profile compared to free TMP. The free drug was released rapidly, in which the cumulative release was up to about 86% over 24 h (Fig. 3). In contrast, TMP-NPs and TAT-TMP-NPs showed the sustained-release behavior, in which about 80% of TMP was released from TMP-NPs and 82% of it from TAT-TMP-NPs within 96 h. These results suggested that the nanoparticles showed the advantage of the controlled-release profile at 37 °C in PBS medium (pH 7.4).

### In vitro hemolysis

**Fig. 4:**
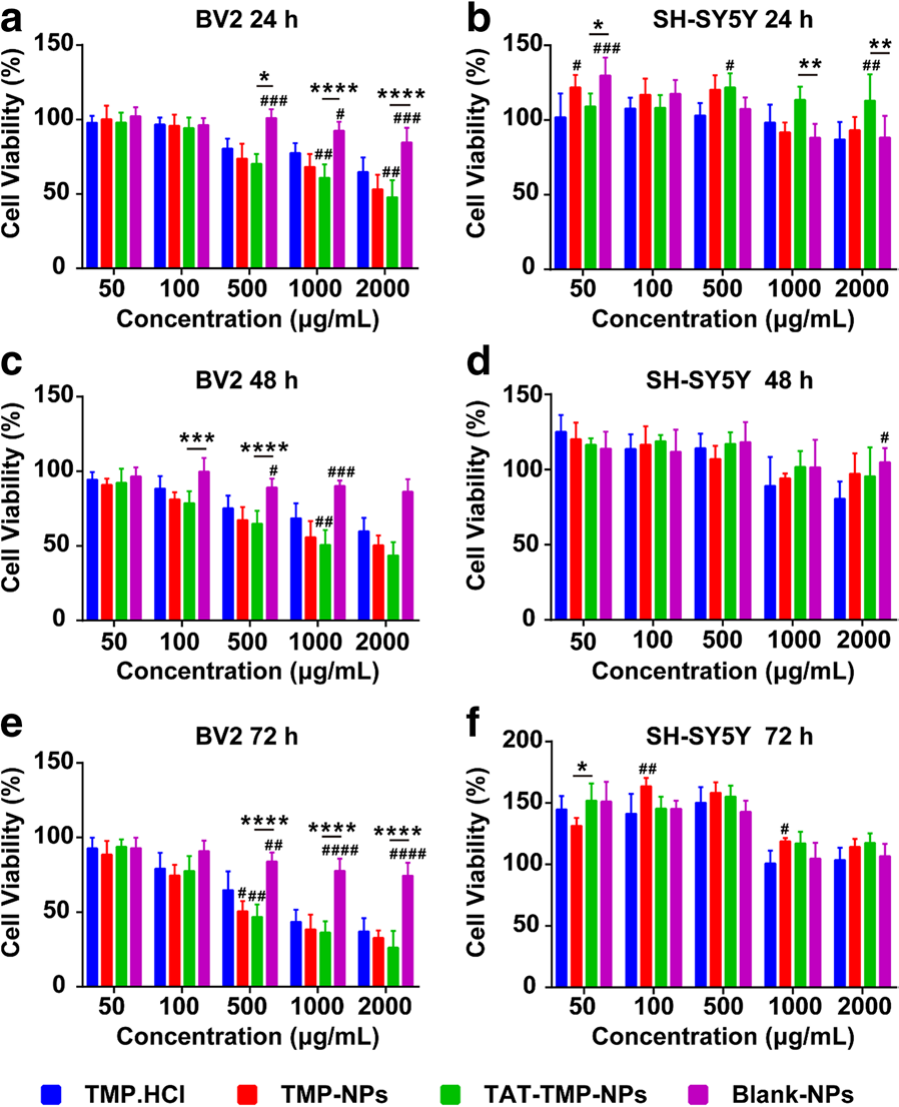
Cytotoxicity assessment after different treatments. Cell viability of BV2 after incubation with different formulations of nanoparticles for 24 h (**a**), 48 h (**c**), 72 h (**e**) and the same operations on SHSH-SY5Y for 24 h (**b**), 48 h (**d**), 72 h (**f**). Data from CCK8 assay were expressed as mean ± standard deviation (n = 5). ANOVA (*P < 0.05, **P < 0.01, ***P < 0.001, ****P < 0.0001) was used to assess the statistical differences between groups. Symbols represented statistical significance of the labeled groups with group of TMP.HCl (^#^*P* < 0.05, ^##^*P* < 0.01, ^###^*P* < 0.001, ^####^*P* < 0.0001)

Results from the hemolytic assay demonstrated that the different hemolytic potential of each administration group when incubated with red blood cells (RBCs) (Fig. 4). As shown in Fig. 4a, the saline group and the administration groups (500 µg/mL) had little or no hemolytic reactions while they were clear and transparent solutions compared with the control group of radio immunoprecipitation assay (RIPA) lysis buffer. The specific analysis was conducted on the hemolysis data of different concentration groups, and the results were shown in Fig. 4b. The erythrocyte hemolysis rate of TMP.HCl, TMP-NPs, and TAT-TMP-NPs showed concentration dependence, and the hemolysis rates of nanoparticles were less than 5%. When the concentration was of 50–100 µg/mL, there was no statistical difference between the groups. The hemolysis rate of TMP.HCl increased significantly at 500 ~ 2,000 µg/mL, and there were statistical differences with other preparation groups (^#^*P* < 0.05, ^##^*P* < 0.01, ^###^*P* < 0.001, ^####^*P* < 0.0001). So, we can assume that the preparation of TAT-TMP-NPs has a low hemolysis reaction and good safety.

### Cytotoxicity

**Fig. 5:**
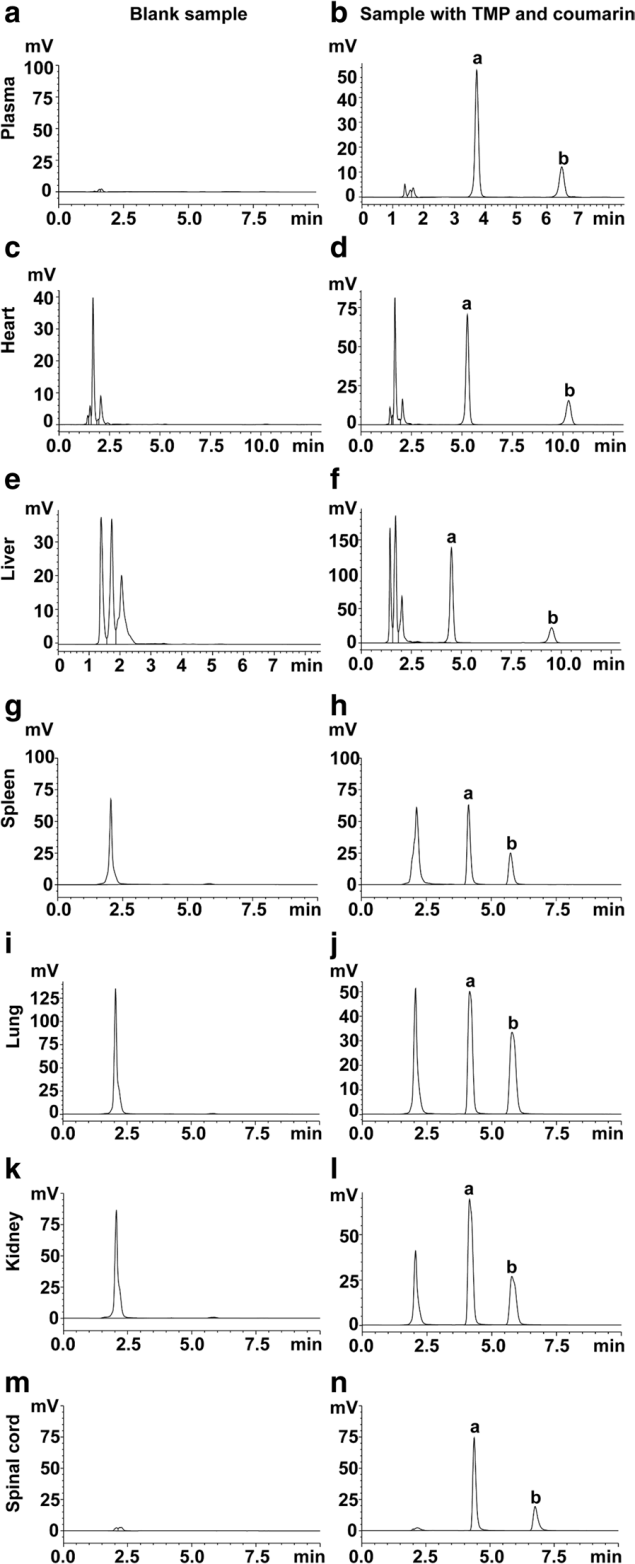
The HPLC chromatograms for determination of TMP (300 µg/mL) and coumarin (internal standard, 10 µg/mL). Plasma: Blank plasma (**a**); plasma spiked with TMP and coumarin (**b**). Heart: blank heart tissue homogenate (**c**); TMP and coumarin spiked in heart tissue homogenate (**d**). Liver: blank liver tissue homogenate (**e**); TMP and coumarin spiked in liver tissue homogenate (**f**). Spleen: blank spleen tissue homogenate (**g**); TMP and coumarin spiked in spleen tissue homogenate (**h**). Lung: blank lung tissue homogenate (**i**); lung tissue homogenate spiked with TMP and coumarin (**j**). Kidney: blank kidney tissue homogenate (**k**); TMP and coumarin spiked in kidney tissue homogenate (**l**). Spinal cord: blank tissue homogenate (**m**); TMP and coumarin spiked in spinal cord tissue homogenate (**n**). a is tetramethylpyrazine, and b is internal standard (coumarin)

As shown in Fig. 5, all the administration groups had low cytotoxicity effects on both BV2 cells and SH-SY5Y cells in the range of 50–100 µg/mL after incubation for 24 h, 48 h, and 72 h. However, compared with TMP.HCl, the cell viabilities of lipopolysaccharide (LPS) -activated BV2 cells were significantly reduced when the concentration of TAT-TMP-NPs and TMP-NPs were 500 µg/mL, 1000 µg/mL and 2000 µg/mL at three time points (^#^*P* < 0.05, ^##^*P* < 0.01) in Fig. 5a, c, e. Meanwhile, after SH-SY5Y cells were co-incubated with 50–500 µg/mL of each administration group for 24 h, 48 h, and 72 h, the viabilities of cells were above 95%. The viabilities of SH-SY5Y decreased slightly when the concentration of administrations increased to 1000– 2000 µg/mL at different time points, but the cell viabilities were still more than 80% in Fig. 5b, d, f, which indicated the groups of TMP.HCl, TMP-NPs, TAT-TMP-NPs, and Blank-NPs had little or no cytotoxicity to SH-SY5Y cells.

In the range of 50–100 µg/mL, LPS-activated BV2 cells and SH-SY5Y cells showed low cytotoxicity in each group. However, as the concentration and incubation time increased, each group showed a concentration-dependent manner on LPS-activated BV2 cells.

### In vivo pharmacokinetics of TAT-TMP-NPs

**Fig. 6:**
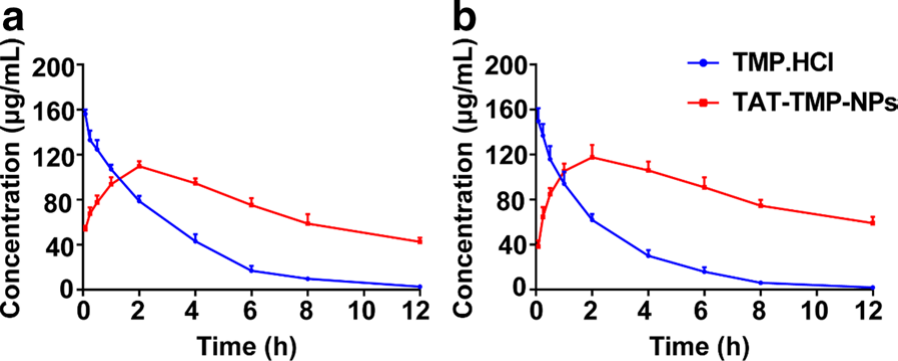
The concentration versus time curve of TMP.HCl and TAT-TMP-NPs in plasma from the healthy rat (**a**) and SCI rat (**b**). Data were expressed as mean ± SD (n = 5)

**Fig. 7:**
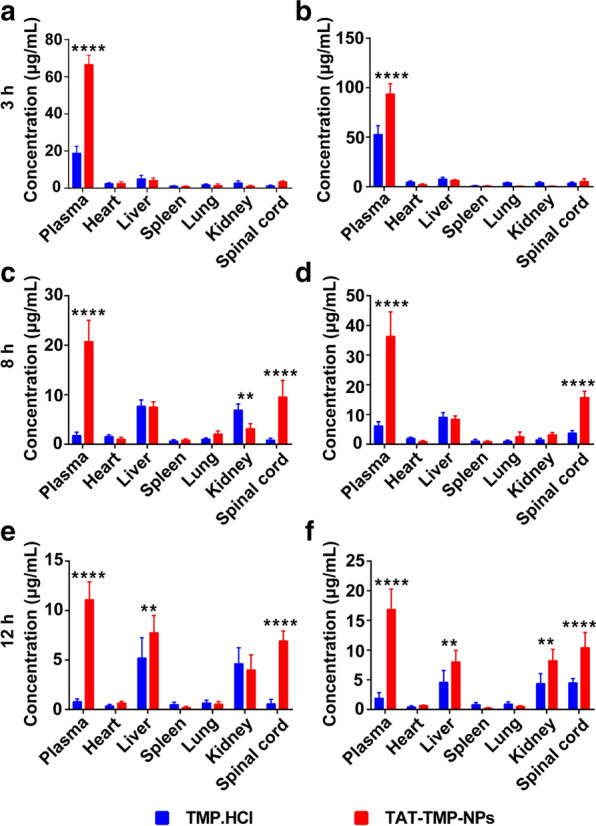
In vivo biodistribution of TMP.HCl and TAT-TMP-NPs in healthy rat and SCI rat. After the healthy rats and SCI rats were administrated with TMP.HCl and TAT-TMP-NPs, these rats were sacrificed and the heart, liver, spleen, lung, kidney and spinal cord were separated at 3 h (**a**, **b**), 8 h (**c**, **d**), and 12 h (**e**, **f**), respectively. Concentration of TMP was measured by HPLC. Data were expressed as mean ± standard deviation (n = 5). ANOVA (**P < 0.01, ****P < 0.0001) was used to assess the statistical differences between groups

In high performance liquid chromatograph (HPLC) detection, the standard curve was linear over the range of 0.25–300 µg/mL (R^2^ = 0.9991) during validation. The relative standard deviation (RSD) ranged from 2.25 to 5.91% for within-day precision of the samples and from 3.11 to 5.65% for between-day precision of the samples. The recovery of the method ranged from 91.94 ± 8.68% to 95.26 ± 2.89%. Figure 6 indicated that the specific mobile phase for the optimum separation of tetramethylpyrazine and coumarin (internal standard) from tissue samples was acetonitrile-water (v/v, 50:50) in less than 10 min. The determination of TMP and coumarin were not interfered by the endogenous substances in plasma and tissue samples. We established a reliable method about TMP content to evaluate the pharmacokinetics of TAT-TMP-NPs in vivo. The mean plasma concentration-time profiles of TMP.HCl and TAT-TMP-NPs in both healthy and SCI groups were shown in Fig. 7. When the rats were treated with the same dose, the release rate and elimination of TAT-TMP-NPs were lower than that of TMP.HCl in both healthy (Fig. 7a) and SCI groups (Fig. 7b). The pharmacokinetics profiles could be explained by the sustained release characteristic of TAT-TMP-NPs mentioned previously.

The pharmacokinetic parameters of TMP.HCl and TAT-TMP-NPs in healthy rats and SCI rats were listed in Tables 3 and 4, which showed differences in the pharmacokinetics for them. The values of T_max_ and C_max_ of TMP.HCl were higher than that of TAT-TMP-NPs, and TMP.HCl exhibited higher fast clearance (CL) than TAT-TMP-NPs. The phenomenon was strongly supported by the significantly increased mean residence time (MRT) of TAT-TMP-NPs in healthy rats and SCI rats, and the parameters suggested that the elimination would not be affected by the pathological state. These results indicated that TAT-TMP-NPs prolonged the systemic circulation time and improved the bioavailability significantly.

**Table 3:** Pharmacokinetic parameters of TMP.HCl and TAT-TMP-NPs in plasma of healthy rats after intravenous injection (n = 5)

Parameters	Unit	TMP.HCl	TAT-TMP-NPs
		Mean ± SD	Mean ± SD
C_max_	µg/mL	156.262 ± 3.380	109.783 ± 4.064
T_max_	h	0.083	2
AUC_0 − t_	µg/mL * h	454.160 ± 27.728	887.623 ± 28.530
MRT	h	2.633 ± 0.142	5.075 ± 0.095
t_1/2_	h	2.066 ± 0.116	7.312 ± 1.916
CL	mL/h/kg	18.200 ± 1.166	6.600 ± 1.020
Vd	mL/kg	54.200 ± 2.040	64.800 ± 5.344

**Table 4:** Pharmacokinetic parameters of TMP.HCl and TAT-TMP-NPs in plasma of SCI rats after intravenous injection (n = 5)

Parameters	Unit	TMP.HCl	TAT-TMP-NPs
		Mean ± SD	Mean ± SD
C_max_	µg/mL	150.957 ± 9.830	120.368 ± 7.118
T_max_	h	0.117 ± 0.067	2.200 ± 0.980
AUC_0 − t_	µg/mL * h	375.002 ± 33.859	1042.315 ± 44.987
MRT	h	2.418 ± 0.148	5.371 ± 0.038
t_1/2_	h	1.982 ± 0.259	8.329 ± 1.813
CL	mL/h/kg	22.000 ± 2.098	4.800 ± 0.748
Vd	mL/kg	63.600 ± 9.047	59.200 ± 6.615

*AUC*_0−t_ area under curve, *MRT* Mean residence time, *t*_1/2_ elimination half life time, *CL* total body clearance from plasma, *Vd* apparent volume of distribution

### Tissue distribution

The results from Fig. 7 showed that TMP concentration in the plasma was the highest one among all the detected tissues at 3 h. The TMP concentration from TAT-TMP-NPs in tissues was found to decrease in the order of plasma > spinal cord > liver > kidney at 8 h, plasma > liver > spinal cord > kidney at 12 h in healthy rats, and plasma > spinal cord > liver > kidney at 12 h in SCI rats, respectively. TMP.HCl was rarely distributed in spinal cord tissue that was significantly different from TAT-TMP-NPs (****P < 0.0001), suggesting the presence of the blood spinal cord barrier could prevent many drugs from crossing this barrier to the site of the spinal cord.

## Discussion

TMP has been proved to have some disadvantages like short half-life, poor water solubility and low bioavailability [22]. In order to solve these problems, this study prepared TMP into the nano-preparations. The results show that the prepared nanoparticles have a spherical structure, uniform particle size, and particle size of less than 200 nm, and could penetrate tissues to the lesion site. The physicochemical properties of nanoparticles, such as small size, large surface area, and flexible chemical compositions or structures that facilitate their use in nanomedicine [23]. Nanoparticles have already advanced the treatment of several challenging conditions including cancer and human immunodeficiency virus (HIV), but it is still possible to induce toxicological results by unexpected ways, so, understanding cell-nanoparticle interactions is critical to developing effective nanosized drug delivery systems [24]. Although nanoparticles materials are prone to toxic and side effects when applied, HSA has been shown to reduce the toxicological side effects of nanoparticles in vitro cytotoxicity experiments, indicating that HSA has potential applicability as a drug carrier. TMP-NPs and free TMP show almost the same cytotoxic activity, indicating that HSA-NPs as a drug carrier do not affect the therapeutic effect of TMP on SCI. The accumulative release in vitro shows that the encapsulated drug had a good sustained-release function, and the construction of the target drug delivery system could extend the treatment time of the drug in vivo. In vivo animals imaging experiments show that TAT-TMP-NPs mainly distributes in the spinal cord and had good organ-targeting properties, which also prove that TAT can increase the membrane penetration rate of TMP. Pharmacokinetic studies show that TAT-TMP-NPs can prolong the half-life of TMP compared with other groups. TAT-TMP-NPs is expected to be an ideal candidate drug with great potential in clinical application. It has been reported that, in addition to changing drug dosage forms to prolong drug action time, the most commonly used strategy is to conjugate the polyethylene glycol (PEG) polymer onto the surface of the nanoparticles which can increases circulation half-life of nanoparticles [25]. In addition to drug therapy, daily nursing of SCI patients is also a major problem in the process of SCI treatment. It is known that spouses of people with SCI have a critical role in assisting the adjustment of the person with the injury, use formal carers to reduce the load of care-giving and adapt family roles to fit with the capabilities of the spinal-injured person [26].

## Conclusions

We have successfully prepared a new delivery system of TAT-TMP-NPs. In vitro hemolysis test and cytotoxicity test showed the safety of drugs. The results of pharmacokinetics showed that the delivery system could prolong the action time of drugs in vivo. The results of animal in vivo imaging demonstrated the targeting ability of TAT-TMP-NPs to spinal cord injury.

## Materials and methods

### Materials

Tetramethylpyrazine (TMP) was purchased from Sigma-Aldrich (St. Louis, MO, USA; purity > 98%). Human serum albumin and 4′,6-diamidino-2-phenylindole (DAPI) were provided by Solarbio (Beijing, China). Roswell Park Memorial Institute-1640 medium (RPMI-1640) was obtained from Hammer Flew (Beijing, China). Fetal bovine serum was purchased from TianHang (Huzhou, China). Trypsin and Alexa Fluor® 555 were provided by Thermo Fisher Scientific (Waltham, MA, USA). Methanol and acetonitrile (HPLC grade) were purchased from Kelong Chemical Reagent Factory (Chengdu, China). ELISA kits were from Shanghai Qiaodu Biotechnology Company (Shanghai, China). TAT-PEG_2000_-chol was synthesized by our laboratory.

### Cell lines and animals

The human neuroblastoma cell line SH-SY5Y and mouse microglial cell line BV2 were purchased from the Shanghai Cell Institute, China Academy of Sciences, and preserved in our laboratory. Both SH-SY5Y and BV2 cells were cultured in RPMI-1640 containing 10% FBS, 100 U/mL penicillin, and 100 mg/mL streptomycin at 37 °C with 5% CO_2_.

Male Sprague–Dawley (SD) rats (200 ± 20 g, n = 110) of specific pathogen-free grade were provided by the Experimental Animal Center of Southwest Medical University (Luzhou, China, SYXK2013-065). All experimental procedures were approved by the Animal Ethics Committee of Southwest Medical University and performed in strict accordance with the management regulations of the Animal Protection and Use Committee of Southwest Medical University.

### Methods

#### Rat model of SCI

SD rats were selected as the model animals, and the modified Allen’s heavy-hitting method was used to establish a reliable SCI model as previously reported [27]. Briefly, after the rats were anesthetized by 10% chloral hydrate (350 mg/kg) and were fixed on the operating table, lamina and transverse processes were exposed and underwent a laminectomy at the T9-T10 vertebra. And then the SCI model was induced by dropping a 10 g hammer freely from a 15 cm height on the exposed dura mater. Successful signs of the SCI model were represented by the presence of twitched, sagged tail, and jerked hind legs. After the wounds were closed aseptically and ampicillin (100 mg/kg) was injected to prevent the wound infection. The bladders of the SCI rats were massaged twice a day to help urinate.

#### Preparation of nanoparticles

The tetramethylpyrazine-loaded nanoparticles (TMP-NPs) or the cell-penetrating peptide TAT modified TMP-NPs (TAT-TMP-NPs) were prepared by the emulsification-dispersion technique as previously reported with modification [28]. Firstly, 30 mg of TMP with or without TAT-PEG_2000_-Chol (30 mg) were dissolved in chloroform as an oil phase; Secondly, HSA 200 mg was dissolved in distilled water to form a 2% protein solution (w/v, 10 mL) and adjusted to pH 4.0 using HCl solution (0.1 mol/L). Thirdly, the oil phase was added into the protein solution, and the resultant mixture was kept for 15 min under shearing at 800 W through an ultrasonic cell disruptor (Scientz-IID, Ningbo, China). Finally, the chloroform was evaporated under vacuum at 40 °C to obtain the organic solvent-free suspension of TAT-TMP-NPs or TMP-NPs.

#### Characterization of nanoparticles

The particle size and PDI, and zeta potential of blank Blank-NPs, TMP-NPs and TAT-TMP-NPs were measured by using a Malvern Zetasizer (Nano ZS90, Malvern Instruments, U.K.). Morphology of nanoparticles was conducted using a transmission electron microscopy (TEM, JEM-100SX, Japan). To determine the encapsulation efficiency (EE) and drug loading (DL) of TMP-NPs and TAT-TMP-NPs, a high-performance liquid chromatography system (HPLC-1260, Agilent, USA) with a UV detector set at 290 nm was used. Measurements were performed on a reversed-phase column (ODS C18, 4.6 × 150 mm, 5 µm, Agilent, USA) at 25 °C. The flow phase was the mixture of methanol and ultrapure water (60:40, v/v) and the flow rate was 1 mL/min, and the sample volume is 10 µL. EE and DL were determined using the following formulae:

$${\text{EE}} (\% ) = {\text{(weight of the drug in nanoparticles)/(weight of total drug)}} \times 100\%.$$

$${\text{DL}} (\% ) = {\text{(weight of the drug in nanoparticles)/(weight of total nanoparticles)}} \times 100\%.$$

#### In vitro release of TMP-NPs and TAT-TMP-NPs

The in vitro release behaviors of TMP from TMP-NPs and TAT-TMP-NPs were investigated using a dialysis method with PBS (pH 7.4, containing 2.5% Poloxamer) as the release medium [29]. Briefly, 4 mL of freshly prepared TMP-NPs, TAT-TMP-NPs, and free TMP were added into dialysis bags (molecular weight cut-off of 3.5 kDa, Millipore), respectively. The dialysis bags were submerged into 60 mL of release medium. The sealed vials were placed in a water bath stirring at a speed of 70 rpm for 96 h at 37 °C. At different intervals of 0.5, 1, 2, 4, 6, 8, 12, 24, 36, 48, 72, and 96 h, 1 mL of the release medium was taken out and replaced with the same volume of fresh medium. The sample was filled to 5 mL with methanol and the concentration of the released TMP from nanoparticles was determined by HPLC as described above.

#### Hemolysis assay

The hemolytic potential of the TAT-TMP-NPs drug delivery system was evaluated through the rat RBCs in vitro. Fresh RBCs obtained from the blood of male SD rats were washed three times with saline and centrifuged at 2,000 rpm for 10 min, then abandoned the supernatant and resuspended RBCs using 10 mL of saline to obtain RBCs suspension (2%, volume ratio). Different concentrations (50, 100, 500, 1,000, 2,000 µg/mL) of TMP, TMP-NPs, TAT-TMP-NPs, and Blank-NPs were incubated with RBCs suspension at a 1:1 ratio for 3 h, taking saline and RIPA as negative and positive controls, respectively. After incubation, the samples were centrifuged at 3,000 rpm for 5 min. 200 µL of the supernatants were obtained to analyze the hemoglobin content using a microplate reader at 576 nm [30]. The hemolysis rate (%) was calculated as the following formula:

$${\text{Hemolysis rate}}(\% ) = ({\text{A}}_{{{\text{sample}}}} - {\text{A}}_{{{\text{negative control}}}} )/({\text{A}}_{{{\text{positive control}}}} - {\text{ A}}_{{{\text{negative control}}}} ) \times 100\%.$$

#### Cytotoxicity

LPS-activated microglial cells BV2, and the healthy human neuroblastoma cells SH-SY5Y were used as model cells. Cell counting kit-8 (CCK8) assay was performed with both LPS-activated BV2 cells and SH-SY5Y cells [31]. Briefly, 1 mL of BV2 cell suspensions (5 × 10^4^ cells/mL) were inoculated into 24-well culture plates, and cultured until cells were completely attached to the bottom of plates. 10 µL of LPS solutions was added and incubated for 4 h to produce the inflammatory activated BV2 cells. Then both LPS-activated BV2 cells and SH-SY5Y cells were respectively seeded in RPMI-1640 culture medium with 10% fetal bovine serum (FBS). After incubation for 24 h, the medium was replaced with the fresh medium containing 100 µL of different concentrations (50, 100, 500, 1,000, 2,000 µg/mL) of TMP, TMP-NPs, TAT-TMP-NPs and Blank-NPs and co-incubated respectively at 37 °C for different time points (24 h, 48 h, 72 h), taking the blank medium as blank controls. Cell viabilities of LPS-activated BV2 and SH-SY5Y were measured by CCK8 assay with the absorbance determined through a microplate reader at the wavelength of 450 nm. Cell viability was calculated using the following formula:

$${\text{Cell viability}}(\% ) = {\text{ }}({\text{A}}_{{{\text{sample}}}} - {\text{A}}_{{{\text{blank}}}} )/({\text{A}}_{{{\text{control}}}} - {\text{A}}_{{{\text{blank}}}} ) \times 100\% .$$

#### In vivo pharmacokinetic study

SD rats were randomly divided into 4 groups (n = 10) following as: Healthy + TMP.HCl, Healthy + TAT-TMP-NPs, SCI + TMP.HCl, SCI + TAT-TMP-NPs. All SD rats were injected intravenously with 0.4 mL of the designed formulations mentioned above as saline, TMP.HCl, TMP-NPs or TAT-TMP-NPs once a day for 7 days, in which the TMP dose was 8.4 mg/kg, as determined by the dose conversion factor for humans and rats and our preliminary experiments. Healthy rats were injected with an equal volume of saline as control.

To get the in vivo pharmacokinetic profile, 1.5 mL of the blood sample was collected from the rat’s postorbital vein at the different intervals of 5 min, 15 min, 30 min, 1 h, 2 h, 4 h, 6 h, 8 h, 12 h after administration. The samples were centrifuged at 6,000 rpm for 5 min and 90 µL of the plasma was mixed with 10 µL of the internal standard of coumarin (10 µg/mL). And then, 0.3 mL of acetonitrile/methanol (5:1, v/v) was added for the process of protein deposition. The resulting solution was vortexed and centrifuged for 10 min at 10,000 rpm. 10 µL of supernatants was injected for analysis by using an HPLC system (1260, Agilent, USA) with a UV detector set at 280 nm. Measurements were performed on a reversed-phase column (ODS C18, 4.6 × 150 mm, 5 µm, Agilent, USA) at 25 °C. The mobile phase was composed of acetonitrile and water (50/50, v/v), and the flow rate was 1.0 mL/min [32, 33]. As were determined, and TMP concentration at each time point in the plasma was calculated by an internal standard curve method to obtain a concentration-time profile. Pharmacokinetics parameters including the maximum plasma concentration (C_max_), time to reach the maximum concentration (T_max_), area under the plasma concentration-time curves (AUC_0 − t_), mean residence time (MRT), plasma elimination half-life (t_1/2_), total body clearance (CL), apparent volume of distribution (Vd) of TMP.HCl and TAT-TMP-NPs in both healthy rats and SCI rats were performed according to the concentration-time profiles by using DAS ver.2.1.1 software.

#### Distribution of TAT-TMP-NPs in SCI model rats

Both the healthy rats and SCI model rats were intravenously administrated with TMP.HCl and TAT-TMP-NPs, respectively. At 3 h, 8 h, and 12 h post-administration, the rats (n = 5 for each time point) in each group were sacrificed, and major organs and tissues including heart, liver, spleen, lung, kidney and spinal cord were rapidly excised and rinsed thoroughly with saline. The tissues were homogenized in ice-cold saline with a hand-held tissue homogenizer and centrifugation at 10,000 rpm for 5 minutes to obtain the supernatant. TMP concentrations in each tissue were determined by the HPLC system mentioned above.

Meanwhile, following the intravenous injection in SCI model rats, the distribution of TAT-TMP-NPs was investigated by the *in vivo* small animal imaging system (Bruker, Fx Pro/FX, USA). Briefly, we firstly prepared DiD-NPs and TAT-DiD-NPs, in which DiD as a kind of hydrophobic infrared fluorescent dye encapsulated into nanoparticles or in free form was used as the tracer (5 µg/rat). The SCI model rats (n = 3) were injected intravenously with saline, free-DiD, DiD-NPs, and TAT-DiD-NPs, taking the healthy rats as control. The rats were respectively anesthetized at 3 h, 8 h, and 12 h post-administration and observed by the imaging systems, the fluorescence distribution of nanoparticles by the *in vivo* small animal imaging system (Bruker, Fx Pro/FX, USA). The images of each rat used the same intensity scale with the same range of minimum and maximum values. Finally, after the last detection time point, the rats were sacrificed. The blood and major tissues including the heart, liver, spleen, lung, kidney, and spinal cord were rapidly collected and observed. The fluorescence intensity of images in each rat was quantified using Image software (NIH Image-Pro Plus 6.0).

### Statistical analysis

All data were compared and analyzed using Graphpad 6.01 statistical software, and the experimental data were represented as the mean standard (SD). Differences between different treatment groups were analyzed using 2-way ANOVA for multiple comparisons. p < 0.05 was considered statistically significant.

## Data Availability

We ensure the authenticity and repeatability of the data obtained by this research. The datasets used and/or analyzed during the current study are available from the corresponding author. All data generated or analyzed during this study are included in this published article. For more information please email our Research Data Team (zhongzhirong@126.com).
